# Impact of Laser Structuring on Medical-Grade Titanium: Surface Characterization and *In Vitro* Evaluation of Osteoblast Attachment

**DOI:** 10.3390/ma13082000

**Published:** 2020-04-24

**Authors:** Kai Borcherding, Dennis Marx, Linda Gätjen, Uwe Specht, Dirk Salz, Karsten Thiel, Britt Wildemann, Ingo Grunwald

**Affiliations:** 1 Department of Adhesive Bonding Technology and Surfaces, Fraunhofer Institute for Manufacturing Technology and Advanced Materials (IFAM), 28359 Bremen, Germany; Dennis.Marx@ifam.fraunhofer.de (D.M.); Linda.Gaetjen@ifam.fraunhofer.de (L.G.); Uwe.Specht@ifam.fraunhofer.de (U.S.); Dirk.Salz@ifam.fraunhofer.de (D.S.); Karsten.Thiel@ifam.fraunhofer.de (K.T.); 2 Julius Wolff Institute, BIH Center for Regenerative Therapies, Charité—Universitätsmedizin Berlin, Corporate Member of Freie Universität Berlin, Humboldt-Universität zu Berlin, and Berlin Institute of Health, 13353 Berlin, Germany; Britt.Wildemann@med.uni-jena.de; 3 Experimental Trauma Surgery, Department of Trauma, Hand and Reconstructive Surgery, University Hospital Jena, 07747 Jena, Germany; 4 Industrial and Environmental Biology, Hochschule Bremen—City University of Applied Sciences, Neustadswall 30, 28199 Bremen, Germany; i.grunwald@hs-bremen.de

**Keywords:** osteointegration, titanium, surface, laser, coating, orthopedics, dental

## Abstract

Improved implant osteointegration offers meaningful potential for orthopedic, spinal, and dental implants. In this study, a laser treatment was used for the structuring of a titanium alloy (Ti6Al4V) surface combined with a titanium dioxide coating, whereby a porous surface was created. The objective was to characterize the pore structure shape, treatment-related metallographic changes, cytocompatibility, and attachment of osteoblast-like cells (MG-63). The treatment generated specific bottleneck pore shapes, offering the potential for the interlocking of osteoblasts within undercuts in the implant surface. The pore dimensions were a bottleneck diameter of 27 µm (SD: 4 µm), an inner pore width of 78 µm (SD: 6 µm), and a pore depth of 129 µm (SD: 8 µm). The introduced energy of the laser changed the metallic structure of the alloy within the heat-affected region (approximately 66 µm) without any indication of a micro cracking formation. The phase of the alloy (microcrystalline alpha + beta) was changed to a martensite alpha phase in the surface region and an alpha + beta phase in the transition region between the pores. The MG-63 cells adhered to the structured titanium surface within 30 min and grew with numerous filopodia over and into the pores over the following days. Cell viability was improved on the structured surface compared to pure titanium, indicating good cytocompatibility. In particular, the demonstrated affinity of MG-63 cells to grow into the pores offers the potential to provide significantly improved implant fixation in further *in vivo* studies.

## 1. Introduction

Titanium and its alloys have been used as an implant material for decades [1,2]. Meanwhile, Kurtz et al. [3] predicted a considerable increase in orthopedic procedures of 0.57 million hip arthroplasties and 3.48 million primary total knee arthroplasties until the year 2030, which will further increase the demand. The phase composition (alpha, beta, or alpha + beta) of titanium alloys [4] must especially be taken into account in the implant design to prevent wear and potential recalls by notified bodies [5]. Even though titanium alloys are currently in clinical use, they contain leachable toxic elements such as vanadium and aluminum, which could be released due to corrosion or wear. In this context, the cytocompatibility [6]—or rather the toxicity [7]—of alloys or the interacting surfaces is one of the critical properties requiring the most attention. To reduce these risks and generate optimal osteointegrative long-term implants, current research focuses on alloy developments [8] or new surface techniques [7] to improve these materials in terms of corrosion and wear resistance, with simultaneous biomechanical stability. A further factor to promote osteointegration is the surface topology, which can promote cell attachment and growth. Hence, a variety of technologies have become available [9]. This approach of adapting surface topographies is not new: Hahn et al. [10] already reported their findings in 1970, and knowledge on this topic has increased in subsequent years [11,12]. Against the background of the rising quantity of, and required improvements for, long-term implants, cost-effective methods are needed to equip mass products (e.g., hip prosthesis) as well as additively manufactured patient-specific implants. In a previous work, a laser structuring technology was developed to provide an opportunity for an antimicrobial equipment of the surface (based on an antibiotic and silver) [13]. The indirect cytocompatibility for that hybrid coating was thereby demonstrated. The objective of the present study is to characterize: a) the pore structure shape, b) the metallographic changes due to the laser treatment, c) the cytocompatibility over time (3 days), and d) the attachment of osteoblast-like cells and their affinity to grow into the pores. 

## 2. Materials and Methods

### 2.1. Titanium Alloy Test Specimens

K-wires and squared plates made of titanium TiAl6V4 (grade 5) were used in this study. The K-wire dimensions were 150 mm in length and 1.0 mm in diameter (mahe medical GmbH, Emmingen-Liptingen, Germany). The square plate dimensions were 2 cm × 2 cm with a 1 mm height (Rocholl GmbH, Aglasterhausen, Germany). The surface roughness of the plates and wires were specified as Ra < 5 µm and Rz < 10 µm.

### 2.2. Laser Treatment and Titanium Dioxide Coating

**Figure 1:**
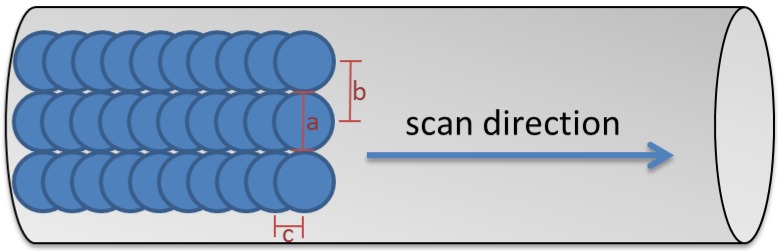
Laser process scheme: spot diameter 107 µm (a), line spacing between the spots 107 µm (b), and distance pulses within a line 53.5 µm (c).

The evaluated structural surface changes were generated using a Q-switched Nd:YAG laser (CL100, Clean Lasersysteme GmbH, Herzogenrath, Germany). The system was operated at a pulse frequency of 100 kHz and a pulse duration of 100 ns. The laser spot was Gaussian-shaped with a diameter of 107 µm (1/e^2^) and a fluence of 5.3 J/cm^2^ in the focal plane. The laser beam was deflected by a 2D scanning galvo mirror system so that the focus meandered over the substrate surface. The line spacing was 107 µm and the distance between the pulses within a line was 53.5 µm (Figure 1). To treat round substrates, they were rotated three times by 120° after the treatment of the side facing the laser.

The cytocompatibility of and attachment of osteoblast-like cells to this surface were compared to the raw material. Complementarily, the laser structure was top-coated with titanium dioxide to mimic the characteristic oxide layer of the raw material. The titanium dioxide layer was deposited after laser structuring. A planar magnetron (VON ARDENNE GmbH, Dresden, Germany) was used for reactive magnetron sputtering in a reduced atmosphere of oxygen (99.998% purity) and argon (99.999% purity) (Linde AG, Pullach, Germany) in an in-house-fabricated coating chamber (Fraunhofer IFAM, Bremen, Germany). 

### 2.3. Micro-Computed Tomography (MicroCT)

The laser’s impact on the inner structure of the titanium, particularly in relation to micro cracking or structural distribution or uniformity, was inspected by micro-computed tomography (µCT) using a µCT scanner (SkyScan 1272; Bruker-µCT, Kontich, Belgium) at 80 kV and 125 μA with an isotropic pixel size of 0.700 μm and a 1 mm aluminum filter. 3D images were reconstructed using the auxiliary CT VOX software version 3.3.0 r1403 and DataViewer Version 1.5.6.2 (Bruker-µCT, Kontich, Belgium).

### 2.4. Electron Microscopy

Surface analyses at the micrometer scale were performed using scanning electron microscopy (SEM) (Phenom XL, Thermo Fisher Scientific, Eindhoven, Netherlands). For the creation of a large-scale surface overview image, SEM pictures were merged using Photoshop CC Version 20.0.5 (Adobe Inc., San José, CA, USA). The characteristic dimensions were measured using ImageJ software (Version 1.52a, Maryland, MD, USA). The crystalline microstructure and the spatial distribution of the elements were assessed on TEM lamellas prepared using the focused ion beam (FIB) technique (Helios 600 dualbeam; FEI, Eindhoven, Netherlands) and analyzed in a transmission electron microscope (Tecnai F20 S-TWIN microscope, FEI, Eindhoven, Netherlands) in scanning mode (STEM) with enhanced diffraction contrast. The elemental distribution was investigated by means of energy-dispersive X-ray spectroscopy (EDX) using an EDAX r-TEM-EDX detector with a resolution of 136 eV measured at Mn Kα.

### 2.5. Metallography

The grain boundaries of the titanium structure were accessed using metallography techniques. The squared plates were cut, sequentially ground with a silicon carbide (SiC) abrasive (grit 80–2500), polished with 0.25 µm colloidal silica suspension, and etched with a solution of hydrofluoric acid and water (3 mL HF, 97 mL H_2_O). The optical micrographs were taken with a DM RX light microscope (Leica, Wetzlar, Germany).

### 2.6. Osteoblast Growth

The MG-63 human cell line (CLS Cell Lines Service GmbH, Eppelheim, Germany) was seeded at 10^5^ cells/mL in McCoy’s 5A medium (with L-glutamine, 10% fetal bovine serum, 100 U penicillin/mL, and 100 μg/mL streptomycin) (Sigma-Aldrich Chemie GmbH, Steinheim, Germany) in 6-well plates on different titanium plates. After incubation for 0.5, 4, 24 h, or 3 days at 37 °C in 5% CO_2_, the cells were fixed with a glutaraldehyde solution (2.5% w/v in PBS) for 30 min at 37 °C. Samples were dehydrated in an ascending ethanol concentration, followed by 30 min in a solution of pure hexamethyldisilazane (HMDS) (abcr GmbH, Kahlsruhe, Germany), pure ethanol (ratio of 1:1), and subsequently in pure HMDS. The samples were removed and dried overnight at room temperature before SEM analysis. 

### 2.7. In Vitro Cytotoxicity

The *in vitro* cytotoxicity was evaluated in accordance with DIN EN ISO 10993-5 guidelines using the WST-1 (4-(3-(4-iodophenyl)-2-(4-nitrophenyl)-2H-5-tetrazolio)-1,3-benzene disulfonate) assay. A volume of 2 mL of MG-63 cell suspension (10^5^ cells/mL in McCoy’s 5A medium) was seeded onto titanium plates and cultivated for 0.5, 4, 24 h, or 3 days at 37 °C in 5% CO_2_. The supernatant was replaced with 2 mL of WST-1 solution in McCoy’s 5A medium and incubated for two hours. The absorption was measured at 450 nm (Mithras LB940, Berthold Technologies, Bad Wildbad, Germany). A cell treatment with 10% hydroxyethyl methacrylate (HEMA) was used as a positive control (cytotoxic) and cells on untreated titanium plates were used as a negative control, n = 3.

### 2.8. Descriptive Statistics

The WST-1 assay data was expressed as interval plots with standard error intervals using the Minitab 18.1 software (Minitab Inc., State College, PA, USA). 

## 3. Results

### 3.1. Microscopic Impact

**Figure 2:**
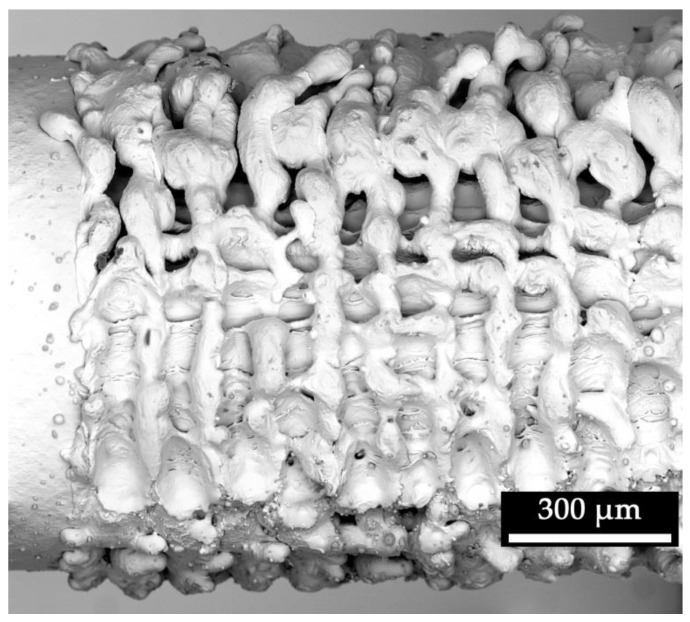
Scanning electron microscopy image of a titanium K-wire surface with (structured surface—right) and without laser treatment (smooth surface—left).

The laser process generated a characteristic, repeating pore structure on the titanium alloy (Figure 2). Due to the laser exposure, the molten titanium increased the diameter of the K-wire from 964 µm (SD: 3 µm) to 1050 µm (SD: 10 µm). The generated typical openings of pores were 70 µm (SD: 17 µm) in length and 49 µm (SD: 5 µm) in width (n = 5), measured within the SEM picture using ImageJ software.

**Figure 3:**
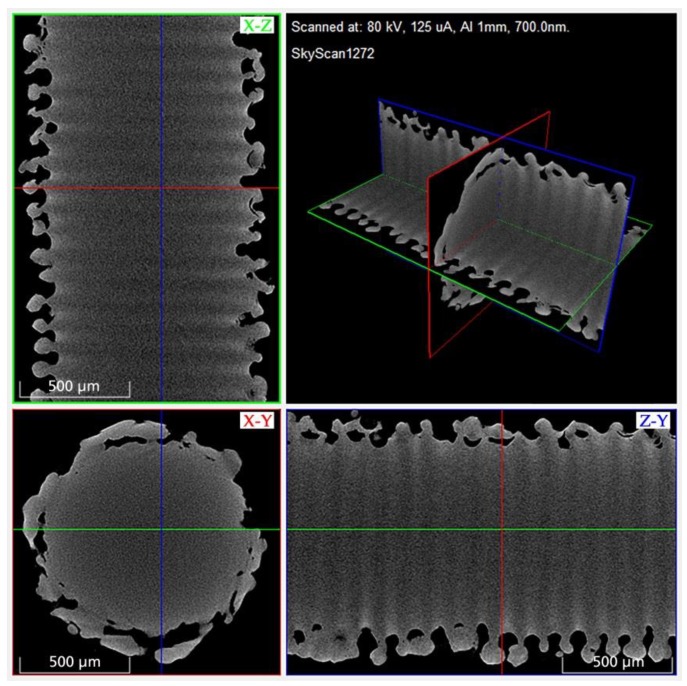
3D rendered micro-computed tomography (µCT) image of a laser-structured K-wire segment for structural analysis (surface, pores, micro cracking).

The µCT analysis confirmed the surface structure appearance in the SEM imaging and provided the possibility for a detailed view and measurement options inside the generated structure (Figure 3). It showed a uniform structural distribution with repetitive pores in an interconnecting pore network. The pore shape is reminiscent of the Greek amphora vase shape: a bulbous-shaped pore with a narrow bottleneck. No indication of micro cracking was found in the whole µCT scan. The generated characteristic pore dimensions were an inner pore width of 78 µm (SD: 6 µm), a bottleneck of 27 µm (SD: 4 µm), and a pore depth of 129 µm (SD: 8 µm) (n = 5). The diameter of 1091 µm (SD: 23 µm) of the K-wire after laser structuring was measured in µCT analysis (n = 5). 

### 3.2. Metallographic Impact

**Figure 4:**
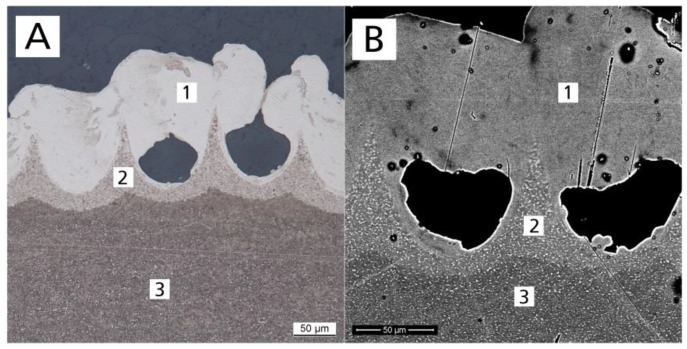
(**A**) Metallographic (etched) optical and (**B**) scanning electron microscope images of a titanium plate cross section after laser treatment, showing the structural composition: (1) surface region, (2) transition region, and (3) substrate region.

Etching with a hydrofluoric acid solution revealed three different regions within the laser structure (Figure 4A) when observed with a light microscope. After etching treatment, light areas were characteristic of an alpha phase and dark areas of a beta phase [14]. The phase of the base alloy (microcrystalline alpha + beta) was transformed to an alpha (enriched) phase of the surface. A peak-shaped region was created between the pores. This region was characterized as a transition region between base alloy (alpha + beta) and surface region (mainly alpha), with increased alpha (lighter than base alloy). SEM confirmed the metallographic findings. The differences in brightness (Figure 4B) indicate differences in the alloy composition of the surface region (Figure 4B(1)), transition region (Figure 4B(2)), and substrate region (Figure 4B(3)). Based on the K-wire diameter before treatment of 964 µm, the diameter in µCT analysis after laser treatment of 1091 µm, and the pore depth of 129 µm, the heat-affected zone was calculated to be approximately 66 µm. 

**Figure 5:**
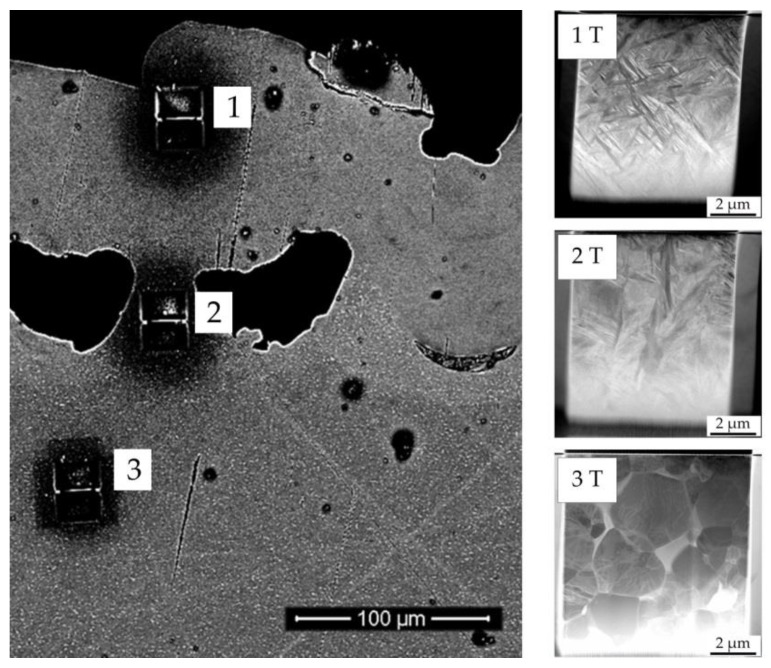
Scanning electron microscope image of a titanium plate surface after laser treatment, presented in cross section. Shown are the focused ion beam preparation sites for the (1) surface, (2) transition, and (3) substrate regions, and the corresponding (scanning) transmission electron microscopy images (1T, 2T, 3T) visualizing the crystalline structure.

For a sub-micrometer analysis in a TEM, from all three regions identified, TEM lamellae were prepared by means of the focused ion beam (FIB) technique. The used enhanced diffraction contrast improved differentiation regarding the crystalline microstructure. The structure of the initial titanium alloy was shown to be in line with expectations from the literature for this type of alloy (globular alpha + beta phase) (Figure 5(3T)). The acicular-shaped structure in the surface region (Figure 5(1T)) indicated a martensite alpha phase. The region in-between (Figure 5(2T)) exhibited mainly acicular-shaped structures, but some globular structures as well, as is expected for the transition region.

**Figure 6:**
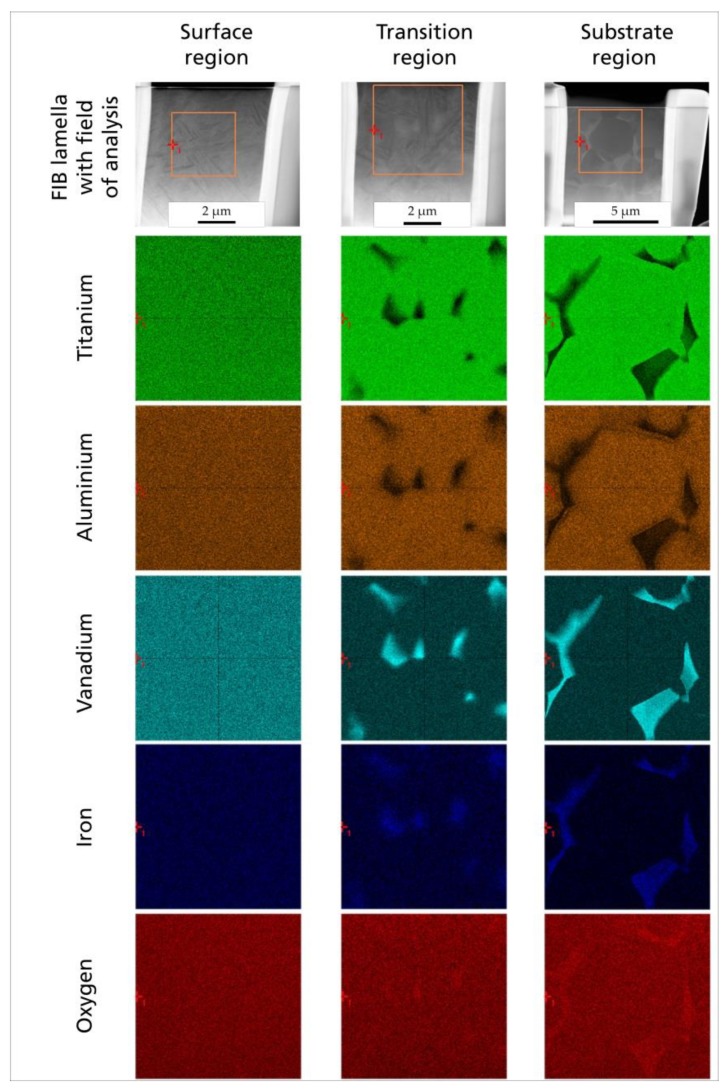
Elemental distribution in each crystalline region of the laser-structured titanium plate (surface, transition, and substrate): STEM image of TEM lamella prepared using focused ion beam (FIB); field of analysis for energy-dispersive X-ray spectroscopy (EDX) and corresponding element mapping for titanium (green), aluminum (brown), vanadium (cyan), iron (blue), and oxygen (red) by EDX.

The EDX mapping revealed that the elemental distribution of the initial crystalline microstructure (substrate region) with its characteristic angular crystal shape and precipitation of mainly vanadium (turquoise) and iron (blue) was changed into a homogeneous distribution (surface region). In between these different microstructures and elemental distributions (transition region), the grain size decreased, and the clear angular shape as well as the elemental precipitation decreased from the substrate to the surface (Figure 6).

### 3.3. Impact on Osteoblasts (In Vitro)

**Figure 7:**
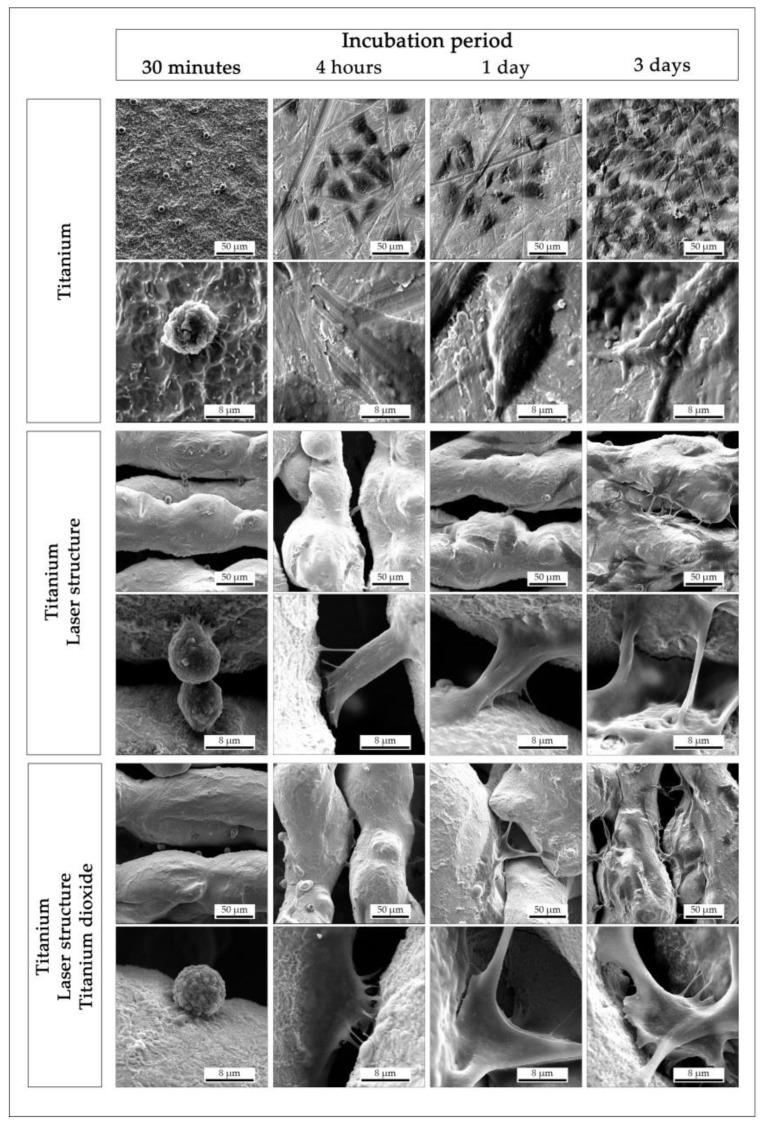
Scanning electron microscope images (overview, detailed) of human osteoblast-like cells (MG-63), visualizing the adhesion over a period of three days for different surface conditions.

Electron microscopy was used to examine the *in vitro* cell morphology during the adhesion and spreading of the cells over time (Figure 7). After 30 min of incubation, the osteoblasts adhered to all surfaces tested. The cell shape was still spherical, but in all experiments, the cells started to extend some filopodia toward the surface. Within four hours of incubation, the osteoblasts presented several filopodia in all directions, whereby the cells on the non-structured titanium surface were almost flat and appeared polygonal. The MG-63 cells on the structured titanium showed a tendency to bridge the pores. After one day (and particularly after three days) of incubation, the affinity to grow over and into the pores with numerous filopodia was observable. In general, no optical difference was noticeable in the cell morphology between the laser-structured titanium with and without further titanium oxide coating. 

**Figure 8:**
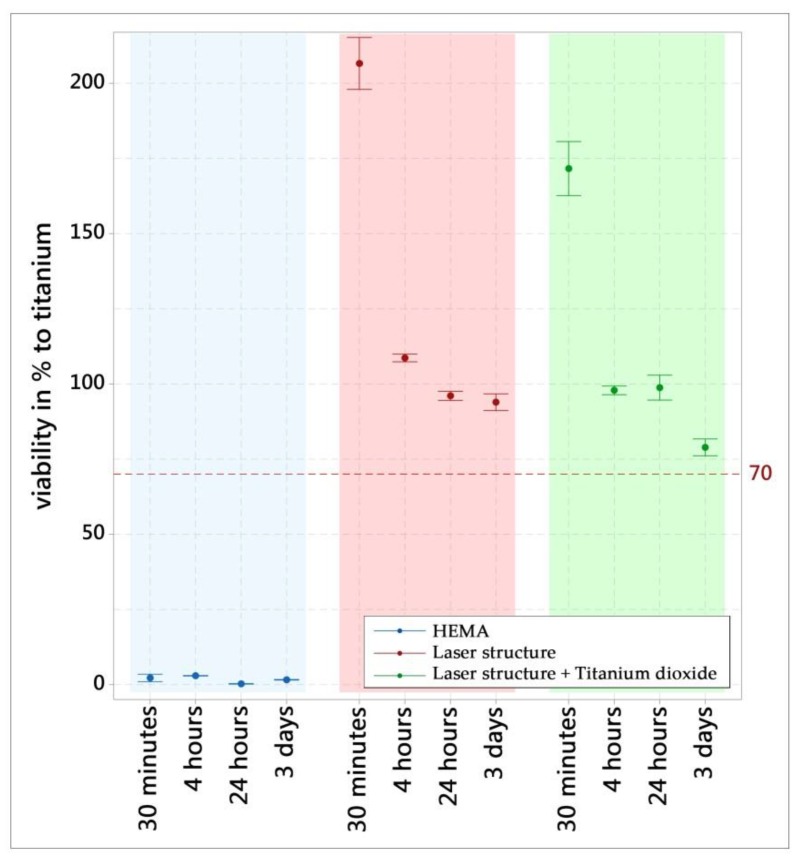
WST-1 assay of the different surfaces in terms of viability (% of titanium surface). The results are displayed as mean values and standard errors (n = 3).

The cell viability for all surface modifications was above the standard recommendation of 70% compared to the negative control (titanium) (Figure 8). The positive control (HEMA) caused a characteristic cytotoxic reaction. In conclusion, the WST-1 results meet the acceptance criteria for *in vitro* cytocompatibility, according to the selected standard. An increase in the absorbance in the WST-1 assay is an indication of an increase in viable cells, which was the case for all the surfaces tested using MG-63. The positive control (cytotoxic) reduced the absorbance of viable cells to (almost) zero. 

## 4. Discussion

The evaluated laser treatment for titanium alloy grade 5 implant surfaces was performed using an Nd:YAG laser, creating a macroscopic undercut without any further preparation of the implant material. As described by Specht et al. [15], porous structures can be generated starting from the nanometer range, as evaluated for a thickness of 150 nm, with this laser type. Furthermore, macroscopic porosity can be generated using an Nd:YAG laser in a reduced oxygen atmosphere, as shown in 2010 by Bandyopadhyay et al. [16], who performed this technique on the basis of computer-aided design data and titanium alloy powder. At present, this technology is known under the term “additive manufacturing”, as summarized in the review by Yuan et al. [17]. In general, the impacts of different laser systems have been evaluated for titanium and titanium alloys with the purpose of triggering the adhesion or proliferation of cells on the surface. A generated surface topography (roughness) can already have a positive impact on bone cells [18]. For example, Iwaszko et al. [19] demonstrated the structuring capacities of a continuous-wave CO_2_ laser treatment for titanium grade 2. Meanwhile, Vorobyev et al. [20] used a Ti:sapphire femtosecond laser to create a variety of surface structures for biomaterial approaches, but again without targeting undercuts for mechanical fixtures for osteointegration. In this study, the energy introduced into the metallic surface by the Nd:YAG laser melted the titanium alloy and induced immediate recrystallization, which changed the metallic phase of the surface region to the alpha phase, as also reported by Khorasani et al. [21]. Furthermore, the surface region exhibited an acicular structure, which indicated a martensite phase similar to the structure shown for the heat treatment, or rather annealing, for grade 5 titanium [22,23]. The EDX element mapping of the surface region showed a uniform distribution of elements. This could be related to the homogeneous distribution of elements within the metallic structure or the thickness of the TEM lamella (successive structure layers). The transition region also exhibited an acicular structure in the TEM imaging, but in contrast to this finding, the EDX element mapping indicated a globular alpha + beta phase, which has been described in the literature for the Ti6Al4V base material [24]. No micro cracking was found in the metallography, electron, or X-ray microscopy analyses, which is in contrast to the results of Zimmermann et al. [25]. This finding could be related to differences in the used laser systems. Compared to the CL250 treatment of Zimmermann et al., the laser treatment with the Cl100 laser was performed with a higher focus and a lower average laser power (Cl100 100 W compared to Cl250 250 W). This results in a narrower embrittled oxygen diffusion zone, which helps to avoid cracks [26]. However, the generated structures and phase compositions can have an impact on fatigue strength [27], whereby residual stress of the material can be reduced and fatigue strength can be increased by laser shock peening [28]. The *in vitro* cytocompatibility of all surfaces was supported in accordance with the ISO 10993-5 standard on the basis of WST-1, which is similar to the findings of Hartjen et al. [29]. The vital osteoblast reaction in adhering and spreading across the surface, and especially the affinity to grow into the pores as well as partially cover them, is a further indication of a cytocompatible surface. This osteoblast behavior is in contrast to the published results regarding osteoblasts that did not spread and acquired a polygonal morphology on rough surfaces [30], whereby it appears that this depends more on the roughness and surface design (peak height and scarped flanks) of the surface, as documented by Ponader et al. [31]. It could also depend on the surface structure, as reported for hole structures in the range of 100 µm that provided good attachment and proliferation of Saos-2 cells [32]. The three-dimensional shape of the generated pores made it difficult to define a characteristic pore, but the measured shape of 70 µm in length and 49 µm in width was within the size range recommended by Ascherl et al. [33] for new bone formation inside of pores, which in turn could provide significantly improved implant fixation through the interlocking of the implant surface and bone.

## 5. Conclusions

A laser treatment that is able to structure standard titanium alloy implants for osteointegration offers meaningful potential for orthopedic, spinal, and dental implants. In particular, the macroscopic undercuts of the structure could provide a mechanical fixation for osteointegration. The vital interaction of osteoblasts (*in vitro*) confirmed this potential, especially due to their affinity to grow over and inside the porous structure. The introduced energy of the laser changed the metallic structure of the alloy within the heat-affected region. The absence of any indication of micro cracking can be positively considered, but the biomechanical impact of this structuring treatment must be reviewed, in particular with regard to potential for crack initiation. Furthermore, as this study was limited to *in vitro* testing, further studies are required to confirm the findings for osteoblast proliferation on and into a laser-structured surface under *in vivo* conditions.
